# Exclusive breastfeeding after home versus hospital birth in primary midwifery care in the Netherlands

**DOI:** 10.1186/s12884-015-0688-8

**Published:** 2015-10-13

**Authors:** TP de Cock, J. Manniën, C. Geerts, T. Klomp, A. de Jonge

**Affiliations:** Department of Midwifery Science, AVAG and the EMGO Institute for Health and Care Research, VU University Medical Centre, Amsterdam, The Netherlands

**Keywords:** Breastfeeding, Midwifery, Birthplace, Primary Health Care, Delivery, Healthcare

## Abstract

**Background:**

Breastfeeding has short-term and long-term health benefits for mother and child. We evaluated in what way birthplace was associated with the rate of exclusive breastfeeding among low risk women who gave birth in midwife-led care and who had expressed the intention to breastfeed.

**Methods:**

We used data from the DELIVER study, which includes pregnant women from twenty midwifery practices across the Netherlands between September 2009 and April 2011. We used data from two questionnaires: one in the third trimester (after 34 weeks) and one after the birth (median 39 days postpartum). Only women who indicated an intention to breastfeed were included in the analyses. Multivariable logistic regression analysis was used to assess the association between birthplace and exclusive breastfeeding, adjusted for relevant confounders.

**Results:**

The exclusive breastfeeding rate was 75.0 % for the 547 women who gave birth at home, and 68.5 % for the 165 women who gave birth in midwife-led care in hospital. The adjusted odds ratio for exclusive breastfeeding after a hospital birth compared to a home birth was 0.79 (95 % CI 0.53–1.18). The most frequently reported reason for not breastfeeding at the time of completing the postpartum questionnaire was ‘my baby was not drinking enough’ (47 %).

**Conclusions:**

In the Netherlands, among low risk women who intended to breastfeed their baby, the breastfeeding success rate did not differ significantly between home and midwife-led hospital births. As breastfeeding has short-term and long-term health benefits for mother and child, women should receive adequate lactation support by healthcare workers during the critical postpartum period, regardless of the place where they give birth.

## Background

Breastfeeding has short-term and long-term health benefits for mother and child. Early benefits for the infant include reduced morbidity from urinary tract, respiratory, gastro-intestinal and middle-ear infections and less atopic illness [1–6]. Breastfeeding offers some protection against the development of diseases in childhood and later in life such as juvenile onset insulin dependent diabetes mellitus [7], raised blood pressure [8, 9], and obesity [10]. Breastfeeding has also been associated with significantly higher scores for cognitive development [11]. Additionally, studies have demonstrated health benefits of breastfeeding to mothers, like enhanced weight loss postpartum [12] and lower incidences of breast cancer [13], ovarian cancer [14] and hip fractures [15].

The World Health Organisation recommends that infants should be exclusively breastfed for the first six months of life to achieve optimal growth, development and health [16]. However, international rates of initiation and duration of breastfeeding are extremely variable between and within countries [17]. The highest incidences (over 90 %) of women who initiated breastfeeding are found in Scandinavia, Eastern Europe and Japan [17]. In the Netherlands in 2000–2002, 78 % of mothers initiated breastfeeding, but after 6 months only 15 % of mothers still provided human milk as the only source of milk feeding [18].

Among those who plan to breastfeed their child, a range of maternal characteristics has been shown to be associated with initiation and/or duration of breastfeeding, varying from personal and structural factors to social factors [17–27]. In addition to these maternal characteristics, breastfeeding initiation and duration might be affected by practices during the intrapartum and very early postnatal period as well as infant characteristics. Previous studies showed positive effects of early mother-newborn skin-to-skin contact [28–32], keeping mother and newborn together [33], and not giving supplemental feeding to breastfed newborns unless medically indicated [33]. Also gestational age at birth [18], birth weight [18, 27], epidural analgesia [27], and oxytocin [27] have been associated with breastfeeding success.

Apart from these known factors predicting breastfeeding success, studies have indicated that the place of birth is likely to play a role as well. The Birthplace study in England showed that babies were significantly more likely to be breastfed at least once if births were planned at home and at freestanding midwifery units compared with planned obstetric unit births [34]. In Canada, more babies were exclusively breastfed at six weeks of age in case of a planned home birth compared to a planned hospital birth [35]. Of all Western countries, the Netherlands has the highest percentage of home births and is therefore ideally suited to study the association between place of birth and breastfeeding rates. A previous Dutch study among 9133 infants in 2000–2002 suggested that home birth was associated with a higher initiation rate as well as longer duration of breastfeeding compared to hospital birth [18]. However, that study might have been biased by the possibility that breastfeeding intention is related to place of birth. It has been suggested that women who intend to breastfeed their child (‘natural’ infant feeding) are probably more likely to prefer a natural, vaginal birth [36].

Similarly, in the Dutch context it is likely that intention to breastfeed is associated with the intention to have a home-birth as opposed to a hospital birth. Restricting studies to women who intend to breastfeed will remove this bias. In order to test whether actual place of birth predicted breastfeeding behaviour, we decided to assess the association in a group of women intending to breastfed their child and who gave birth in a low risk setting, under the supervision of a primary care midwife, either at home or in hospital, so as to control for the various potential confounders which are associated with secondary care births.

## Methods

In the Netherlands, midwives in primary care provide care to low risk women. These are women with a singleton pregnancy of a foetus in cephalic presentation who do not have any medical or obstetric risk factors that are an indication for secondary care, such as previous caesarean section, and who start labour spontaneously between 37 and 42 weeks. If complications or risk factors occur during pregnancy, labour or after birth, women are referred to secondary care, i.e. obstetrician-led care in hospital units. After referral, women may receive care from clinical midwives, obstetricians, obstetric registrars, and obstetric nurses, under the final responsibility of an obstetrician. Midwives refer women if they have an indication as laid out in the obstetric indication list [37], which is revised regularly by a multidisciplinary team. Obstetric interventions such as electronic foetal monitoring, augmentation, and pharmacological pain relief including epidural analgesia do not take place in midwife-led care. Women who are low risk at the onset of labour can choose to give birth at home or in hospital, assisted by their primary care midwife.

### Design

For estimating the association between birthplace and breastfeeding, we used data from the Dutch DELIVER study; DELIVER is a Dutch acronym for data primary care midwifery (Data EersteLIjns VERloskunde). The DELIVER study is a multicentre prospective dynamic cohort study that aimed to evaluate the quality, organisation and accessibility of primary midwifery care in the Netherlands. The methods of the DELIVER study have been described in detail elsewhere [38]. In short, the dynamic cohort consisted of clients who had completed up to three questionnaires between their first prenatal appointment and six weeks postpartum. The first questionnaire was completed before 35 weeks gestation (Q1), the second between 35 weeks gestation and birth (Q2), and the third on average 6 weeks postpartum (Q3). Pregnant women were recruited between September 2009 and April 2011, by midwives from 20 participating midwifery practices spread all over the country. These midwifery practices were selected according to three stratification criteria: region (north, east, south or west), urbanisation level (urban or rural area) and practice type (dual or group practice). For each participating client, questionnaire data were linked to data from the national Netherlands Perinatal Registry and the electronic client record in the midwifery practices by means of unique anonymous client and midwifery practice identifiers.

### Study population

In the DELIVER study, over 14,000 clients were invited to participate and the response rate for at least one questionnaire was 62 %. Comparison with the national population revealed that the total DELIVER study population is representative for parity (nulliparous: 46 % our data versus 47 % national) and age (between 26–35 years: 73 % vs 69 %), but comprises more highly educated women (49 % vs 42 %) and fewer ethnic minority women (16 % vs 25 %) [38].

**Fig. 1:**
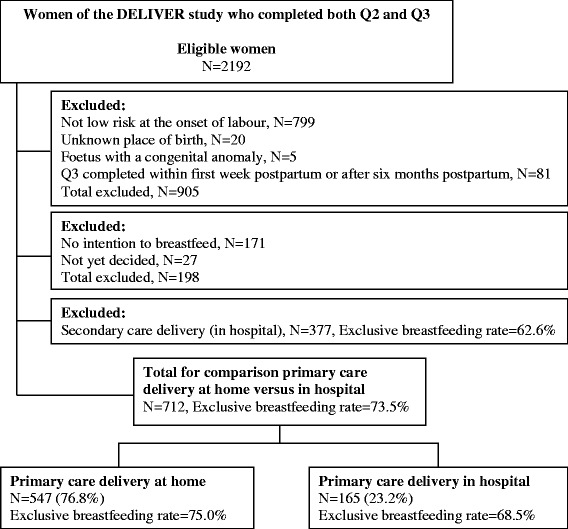
Flow chart of the study population

For this study, we selected women who completed both Q2 and Q3 and were low risk and in primary care at the onset of labour. Additionally, women were excluded if place of birth was unknown, their child had a congenital anomaly, they completed Q3 in the first week postpartum (breastfeeding needed to be established for at least a week) or after six months postpartum (i.e. it is recommended to breastfeed for at least six months). For the analysis of the association between place of birth and rate of exclusive breastfeeding we excluded women who gave birth in hospital under the supervision of an obstetrician (i.e. secondary care) because this is a heterogeneous group very different from the group of women who gave birth in primary care and in which a wide diversity of factors can be present which may be relevant to breastfeeding. It was impossible within this study to account for all potential confounding factors in secondary care which may affect the breastfeeding success rate. Furthermore, we selected only women who had stated the intention to breastfeed in Q2. Figure 1 shows a flow chart of the study population.

### Place of birth and breastfeeding

In Q2, women were asked whether they had the intention to breastfeed their child, and to give reasons if they had no intention to do so. In Q3, we asked women whether they had the opportunity to breastfeed their baby within one hour after birth and to give reasons if they had not. Also in Q3, we asked women whether they were breastfeeding their baby at the moment with the following answering options: ‘Yes, my baby is exclusively breastfed’, ‘Yes, and I also supplement with formula’, ‘No, I tried breastfeeding but stopped after several days/weeks’ or ‘No, I chose not to breastfeed at all’. We asked women for the primary reason for not breastfeeding their baby at the time of completion of Q3. Our primary outcome was the rate of exclusive breastfeeding at the time of completion of Q3, compared to all other women.

Data concerning care at the onset of labour and actual place of birth were extracted from the national Netherlands Perinatal Registry. Place of birth was categorized into home birth and hospital birth, both under the supervision of a primary care midwife (i.e. primary care).

### Covariates

Several demographic and peripartum factors were considered to be potential explanatory or confounding variables for the association between place of birth and the rate of exclusive breastfeeding, namely parity (nulliparous, parous) [18, 19, 22, 25, 27], education level (low/medium, high) [18, 20, 22, 25, 26], education level of partner (low/medium, high) [18], ethnic background (Dutch native/other western, non-western) [26], age (≤24 years, 25–34 years, ≥35 years) [20, 26], smoking before or during pregnancy (yes, no) [18], and birth weight (≤3000 g, 3001–3500 g, ≥3501 g) [18, 27]. Level of education was classified into low/medium (up to higher-level secondary education or vocational education) and high (equivalent to bachelor degree or higher). Classification of ethnic background into Dutch native/other Western and non-Western was according to the definition of Statistics Netherlands [39]. Women are considered ‘Dutch native’ when both of their parents are born in the Netherlands, ‘Western’ when at least one of their parents is born in Europe (excluding Turkey), North America, Oceania, Indonesia or Japan, and ‘non-Western’ when at least one of their parents is born in Asia (excluding Indonesia and Japan), Africa, Latin America or Turkey.

Furthermore, early opportunity to breastfeed, i.e. attempt in first hour postpartum (yes, no) [31], prenatal education on breastfeeding (yes, no) [17], postnatal consult with breastfeeding counsellor (yes, no) [29, 40], and skin-to-skin contact in first hour postpartum (yes, no) [28–32] have been associated with breastfeeding initiation and duration.

### Data analyses

Demographic, pregnancy related and peripartum characteristics as well as infant feeding practices of our study population of low risk women who intended to breastfeed and gave birth in primary care were presented by birthplace. Univariable logistic regression analysis was carried out to determine the association between place of birth and exclusive breastfeeding. We also looked at reported reasons for not having had the opportunity to breastfeed within an hour after birth and reasons for not breastfeeding (any more) at the time of completing Q3. Multivariable analysis was conducted to adjust the association between place of birth and the rate of exclusive breastfeeding for demographic and pregnancy related confounders. In the secondary analysis we controlled the results additionally for peripartum factors associated with breastfeeding initiation and duration (prenatal breastfeeding education, early opportunity to breastfeed, early skin-to-skin contact, postnatal breastfeeding consult). The odds ratio (OR) and the corresponding 95 % confidence interval (95 % CI) were used to summarise the strength of the association between place of birth and exclusive breastfeeding. The level of statistical significance for the study was set at *p* < .05. In both the univariable and multivariable logistic regression analyses, the hierarchical nature of the data, i.e. clients (level 1) nested within midwifery practices (level 2), was taken into account by means of multilevel analyses. If the multilevel model resulted in a significantly better fit than the ordinary univariable or multivariable model, the former was preferred and presented. All analyses were conducted in IBM SPSS statistical software program (version 20.0), except for the multilevel analyses, which were conducted in Stata (version 10). We excluded missing data because they were less than 5 % for all variables.

### Ethics

This study was undertaken as part of the DELIVER study, for which the Medical Ethics Committee of the VU University Medical Centre (Amsterdam) provided ethical approval (WC 008–100). Before participation, informed consent was obtained from all participants. Client participation was voluntary and clients could withdraw at any time from the study.

## Results

### Study population and breastfeeding intention

A flow chart of the study population is presented in Fig. 1. A total of 171 women were excluded because they did not have the intention to breastfeed their child. The most frequently reported reasons for this were: ‘I had a bad experience with breastfeeding’ (44 %), ‘I want my partner to be able to feed the baby too’ (42 %), ‘Formula-feeding is easier’ (28 %), ‘medical reasons (e.g. previous breast surgery, medication)’ (16 %).

The characteristics of the study population are presented in Table 1, stratified by place of birth. Of all 712 included women, 547 (76.8 %) women gave birth at home and 165 (23.2 %) gave birth in hospital. Only small differences in characteristics were observed between women who gave birth at home and those who gave birth in hospital, which were not statistically significant.

**Table 1:** Characteristics of the study population (*N* = 712)

	Home birth		Hospital birth		
Characteristic	*N*	(%)	*N*	(%)	*P* value
*Total*	547	(76.8)	165	(23.2)	
*Ethnic background*					.15
Dutch/other western	529	96.9	154	94.5	
Non-western	17	3.1	9	5.5	
Missing	1		2		
*Education level woman*					.34
Low/medium	203	37.1	68	41.2	
High	344	62.9	97	58.8	
*Education level partner*					.42
Low/medium	263	48.5	85	52.1	
High	279	51.5	78	47.9	
Missing	5		2		
*Age*					.35
≤24 years	32	5.9	8	4.8	
25–34 years	422	77.1	121	73.3	
≥35 years	93	17.0	36	21.8	
*Smoking before or during pregnancy*					.31
Yes	80	14.6	19	11.5	
No	467	85.4	146	88.5	
*Parity*					.24
Nulliparous	172	31.4	60	36.4	
Parous	375	68.6	105	63.6	
*Birth weight*					.97
≤3000 g	36	6.6	11	6.7	
3001–3500 g	195	35.6	57	34.5	
≥3501 g	316	57.8	97	58.8	
Missing	0		0		
*Breastfeeding education prenatally*					.86
Yes	44	8.0	14	8.5	
No	503	92.0	151	91.5	
*Skin-to-skin contact first hour*					.14
Yes	545	99.8	163	98.8	
No	1	0.2	2	1.2	
Missing	1		0		
*Early opportunity to breastfeed*					.16
Yes	525	96.0	154	93.3	
No	22	4.0	11	6.7	
*Postnatal consultation with breastfeeding counsellor*					.37
Yes	65	11.9	24	14.5	
No	482	88.1	141	85.5	

This timing was similar among different birthplaces; the median timing of Q3 was 39 days (P25 = 27 days, P75 = 56 days) for the women who gave birth at home and 40 days (P25 = 26 days, P75 = 52 days) for those who gave birth in hospital.

### Breastfeeding practices

In Table 1 it is shown that of the 712 women, 679 (95.4 %) had the opportunity to breastfeed their baby within one hour postpartum. Reported reasons for not having had the opportunity to breastfeed within an hour after birth are shown in Table 2, which involved 22 (4.0 %) of the women who gave birth at home and 11 (6.7 %) of the women who gave birth in hospital.

**Table 2:** Reasons for not having had the opportunity to breastfeed within an hour after delivery (33 women gave 34 reasons)

Reason	Home birth (*N* = 23)	Hospital birth (*N* = 11)
Complications with the baby	2	5
Complications with myself	11	3
I did not want to breastfeed	1	1
I don’t know	1	1
Other reasons, namely ….	8	1
* … Prolonged suturing*	*4*	*0*
* … Long third stage of labour*	*3*	*0*
* … I did not want to breastfeed yet*	*0*	*1*
* … The midwife was not so quick*	*1*	*0*

At completion of Q3, a total of 523 (73.5 %) babies were breastfed exclusively, 58 (8.1 %) were breastfed in combination with formula feeding, 120 (16.9 %) were breastfed during the first days or weeks but not anymore, and 11 (1.5 %) were never breastfed (Table 3). The rate of women who exclusively breastfed their child was slightly higher among those who gave birth at home (75.0 %) than among those who gave birth in hospital (68.5 %), but this difference was not statistically significant (OR 0.73, 95 % CI 0.50–1.06).

**Table 3:** Infant feeding practices according to place of birth (*N* = 712)

Feeding practice	Home birth (*N* = 547)	Hospital birth (*N* = 165)	Total (*N* = 712)
Exclusive BF, *N* (%)	410 (75.0)	113 (68.5)	523 (73.5)
Not exclusive BF, *N* (%)	137 (25.0)	52 (31.5)	189 (26.5)
BF + FF, *N* (%)	40 (7.3)	18 (10.9)	58 (8.1)
Now FF, previously BF, *N* (%)	88 (16.1)	32 (19.4)	120 (16.9)
Never BF, only FF, *N* (%)	9 (1.6)	2 (1.2)	11 (1.5)
Crude OR (95 % CI)	1 (reference)	0.73 (0.50–1.06)	
Adjusted OR (95 % CI)^a,b^	1 (reference)	0.79 (0.53–1.18)	
Adjusted OR (95 % CI)^a,c^	1 (reference)	0.80 (0.54–1.19)	

*BF* Breastfeeding, *FF* Formula feeding, *OR* Odds ratio, *CI* Confidence interval

^a^10 cases with a missing value for any of the confounders were removed

^b^Adjusted for parity, age, education level, education level partner, ethnicity, smoking, birth weight

^c^Adjusted for b AND peripartum factors associated with breastfeeding initiation and duration (prenatal breastfeeding education, early opportunity to breastfeed, early skin-to-skin contact, postnatal breastfeeding consult)

Table 3 shows that, within our study population of low risk women who planned to breastfeed and gave birth in midwife-led care, the breastfeeding success rate was not significantly lower after a hospital birth than after a home birth when adjusting for demographic and pregnancy related confounders (OR 0.79, 95 % CI 0.53–1.18). Additionally controlling for relevant peripartum factors (prenatal breastfeeding education, early opportunity to breastfeed, early skin-to-skin contact, postnatal breastfeeding consult) could not explain the small difference in breastfeeding success rate between midwife-led home and hospital births; i.e. the ORs when all four factors were added to the model hardly changed (OR 0.80, 95 % CI 0.54–1.19).

Reported reasons for not breastfeeding at time of completing Q3 (*N* = 131) are presented in Table 4. The most frequently reported reason was ‘my baby was not drinking enough’ (*N* = 61, 47 %).

**Table 4:** Reasons for not breastfeeding at the time of completing Q3 (*N* = 131)

	Home birth		Hospital birth	
	Stopped BF (*N* = 88)	Never BF (*N* = 9)	Stopped BF (*N* = 32)	Never BF (*N* = 2)
My baby was not drinking enough	41	1	18	1
BF hurts	10		2	
BF is tiring	7		1	
Medical reason (s)	6	2	1	
Bad experience with BF	5	2	1	
BF is difficult to combine with my job	3			
FF is easier		2		
With FF, my partner is able to feed the baby		1		1
BF hurts	1	1		
Other, namely ….	13		8	
* …. Mastitis*	*4*		*3*	
* …. Other reasons*	*9*		*5*	
Missing	2		1	

*BF* Breastfeeding, *FF* Formula feeding

## Discussion

As far as we know, this is the first study that focused on the effect of place of birth on successful breastfeeding among women who intended to breastfeed. Previous studies in England, Canada and the Netherlands found an association between (planned) place of birth and breastfeeding initiation and duration among low risk women [18, 34, 35]. In our study, women who gave birth at home showed a slightly higher success rate for exclusive breastfeeding around 5.5 weeks postpartum than women who gave birth in hospital (both in primary care), but this difference was not statistically significant.

These findings give further nuance to previous reports in the literature that indicate place of birth as a factor in breastfeeding success [18, 34, 35]. Place of birth was not a significant factor in breastfeeding success in our study population of women who gave birth in primary care and who intended to breastfeed. This difference in results supports the suggestion that breastfeeding intention is associated with (planned) place of birth, i.e. women who intend to breastfeed their child (‘natural’ infant feeding) probably more often prefer a home birth (‘natural’ birth) as opposed to a more medicalised hospital birth. The difference in results could also be partly due to differences in care systems between countries. In the Netherlands, a primary care midwife is responsible for intrapartum care of low risk women. If a woman gives birth in hospital, the midwife only collaborates with a nurse and has no other labouring women to look after. In other countries, labour wards might be busier and low risk women who give birth in hospital are often not supervised by their own primary care midwife but by hospital staff who are often also responsible for the care of other women at the same time. In those conditions, the time to actively provide environmental conditions conducive to promoting mother-to-infant bonding, including lactation support during the postpartum period to all women, might be limited [41]. Hence, in other countries the breastfeeding success rate among low risk women might actually be higher after home births compared to hospital births, and our results might not be applicable to countries with different maternity care systems.

In our study population of women who intended to breastfeed, 73.5 % breastfed their baby exclusively at completion of the questionnaire (median 5.5 weeks postpartum). This breastfeeding success rate is low considering the WHO recommendation to breastfeed all infants exclusively for at least six months. Our rate is somewhat lower than the rate reported of women in Norway [42], but higher than rates in several other countries such as England [43] and China [44]. However, these numbers are difficult to compare with our study as we included only women who intended to breastfeed (about 84 % of the total DELIVER study population), which are more likely to actually breastfeed. Previous studies showed that structured antenatal and postpartum breastfeeding education and extra support are effective means of achieving breastfeeding success [17, 29, 40, 45]. A relatively small number of women in our study followed a course on breastfeeding during pregnancy and/or consulted a breastfeeding counsellor postnatally. This relatively low uptake seems to highlight an area for improvement with regard to optimising breastfeeding success rates in the Netherlands.

This study has a few limitations. Firstly, questions about infant feeding practice were only asked once postpartum and the participants completed the questionnaire at different time points, i.e. between 1 week and 6 months postpartum. However, the timing of completion of the questionnaire did not differ according to birthplace and therefore it is unlikely that this has influenced our results. Secondly, limited information was available on self-confidence, self-esteem, coping capacity and social health, nor on the amount of social and professional support that women received, while previous studies showed that those factors might also influence the initiation and/or duration of breastfeeding [21, 24, 26]. It is recommended in future research to account for psychosocial factors in both (planned) place of birth as well as breastfeeding success.

A strength of our study was the high number of home births in the study population, because that provided an opportunity to reliably assess a possible difference in breastfeeding success between home and hospital births. Another strength was that information was available on many socio-demographic factors as well as on pregnancy and birth related factors. Therefore, we were able to adjust for most of the known factors that potentially confound the association between place of birth and infant feeding practice. In addition, we accounted for clustering of women within midwifery practices.

In our study we already identified some reasons for not being able to breastfeed the child directly after birth or in the postpartum period. However, to better understand factors that influence the exclusive breastfeeding success rate in the Netherlands, it is recommended that a larger study be carried out with the assessment of infant feeding practices at several time points postpartum and more accurate assessment of the actual support women receive intra- and postpartum by health care workers at home and in hospital (e.g. early skin-to-skin contact, keeping mother and newborn together, early initiation of breastfeeding, and not giving supplemental feeding unless medically indicated). This could give insight into whether and how the breastfeeding success rate in the Netherlands can be improved and in what way the place of birth plays a role in breastfeeding success.

## Conclusion

Our study showed that in the Netherlands, among low risk pregnant women in midwife-led care who intended to breastfeed their baby, the breastfeeding success rate did not differ significantly between home and hospital births. As breastfeeding has short-term and long-term health benefits for mother and child, women should receive adequate lactation support by health care workers during the critical postpartum period, regardless of the place where they give birth.
